# Occurrence of Antibodies against SARS-CoV-2 in the Domestic Cat Population of Germany

**DOI:** 10.3390/vaccines8040772

**Published:** 2020-12-17

**Authors:** Anna Michelitsch, Donata Hoffmann, Kerstin Wernike, Martin Beer

**Affiliations:** Institute of Diagnostic Virology, Friedrich-Loeffler-Institut, 17493 Greifswald–Insel Riems, Germany; anna.michelitsch@fli.de (A.M.); donata.hoffmann@fli.de (D.H.)

**Keywords:** SARS-CoV-2, domestic cat, sero-prevalence, COVID-19, diagnostics, serology, felines, Germany

## Abstract

Domestic cats (*Felis catus*) are popular companion animals that live in close contact with their human owners. Therefore, the risk of a trans-species spreading event between domestic cats and humans is ever-present. Shortly after the emergence of the severe acute respiratory syndrome coronavirus 2 (SARS-CoV-2) and its rapid spread around the world, the role of domestic cats in the transmission cycle was questioned. In the present study, the first large-scale survey of antibody occurrence in the domestic cat population in Germany was conducted, in order to assess the incidence of naturally occurring human to cat transmission of SARS-CoV-2. A total of 920 serum samples, which were collected from April to September of 2020, were screened by an indirect multispecies ELISA. Positive samples were verified using an indirect immunofluorescence test (iIFT) and additionally tested for neutralizing antibodies. Furthermore, serum samples were screened for antibodies against feline coronavirus (FCoV), in order to rule out cross-reactivity in the described test systems. Overall, 0.69% (6/920) of serum samples were found to be positive for antibodies against SARS-CoV-2 by ELISA and iIFT. Two of these reactive sera also displayed neutralizing antibodies. No cross-reactivity with FCoV-specific antibodies was observed. The finding of SARS-CoV-2 antibody-positive serum samples in the domestic cat population of Germany, during a period when the incidence of human infection in the country was still rather low, indicates that human-to-cat transmission of SARS-CoV-2 happens, but there is no indication of SARS-CoV-2 circulation in cats.

## 1. Introduction

The domestic cat (*Felis catus*) is a synanthropic species [1] that is found all over the world in the form of feral populations, as well as a popular domestic pet. The amount of contact between domestic cats and humans varies, but can be as close as sleeping in one bed and licking the owner’s face [2]. In particular, domestic cats that are also roaming around in the neighborhood can bring pathogens into the domestic area of humans [3]. Therefore, it is not surprising that domestic cats are a potential risk factor in the spread of zoonotic diseases that is too often overlooked [4].

As the severe acute respiratory syndrome coronavirus 2 (SARS-CoV-2) emerged at the end of December 2019 [5] and became a global pandemic, the role of domestic pets in the spread of the virus was questioned [6]. The public debate was controversial and led to the killing and abandonment of thousands of pets, without sound scientific evidence [7]. Among popular pet species, domestic cats seemed to be a factor for virus transmission since they already displayed corresponding infections with their human owners in the SARS-CoV epidemic back in 2003 [8]. Experimental infections of domestic cats demonstrated that they are susceptible to SARS-CoV-2 infection, spreading the virus to co-housed contact animals [6,9,10,11]. Additionally, small-scale serological investigations of domestic cat populations from affected areas showed that antibodies against SARS-CoV-2 are detectable in domestic cats [12] and that the prevalence correlates with human infections [13]. The first reports of naturally infected cats have been presented for various countries, further proving the natural occurrence of SARS-CoV-2 infections in domestic cats [14,15,16]. It can be assumed that transmission from humans to cats can occur, supported by sequence analysis of viral genomes from domestic cats and humans, which has revealed a high degree of sequence conservation. Single nucleotide polymorphisms in the viral whole genome sequence of cats in comparison to the original Wuhan_Hu-1 reference sequence are also frequently found in genome sequences of SARS-CoV-2 that originated from humans [17]. Overall, the transmission from humans to cats seems to be an occasional event that does not always take place, as nine domestic cats that were monitored during their owner’s infection with SARS-CoV-2 did not acquire an infection themselves [18].

In this investigation, we conducted a first survey of antibodies against SARS-CoV-2 in the domestic cat population of Germany. Additionally, samples were tested for antibodies against feline coronavirus (FCoV)—a coronavirus that is found in domestic cat populations worldwide [19]—to rule out any serological cross-reactivity in the applied testing methods. The goal of this study was to contribute data to the scientific community, in order to enable a better assessment of the frequency of natural human-to-cat SARS-CoV-2 transmission events.

## 2. Materials and Methods

### 2.1. Serum Samples

**Figure 1:**
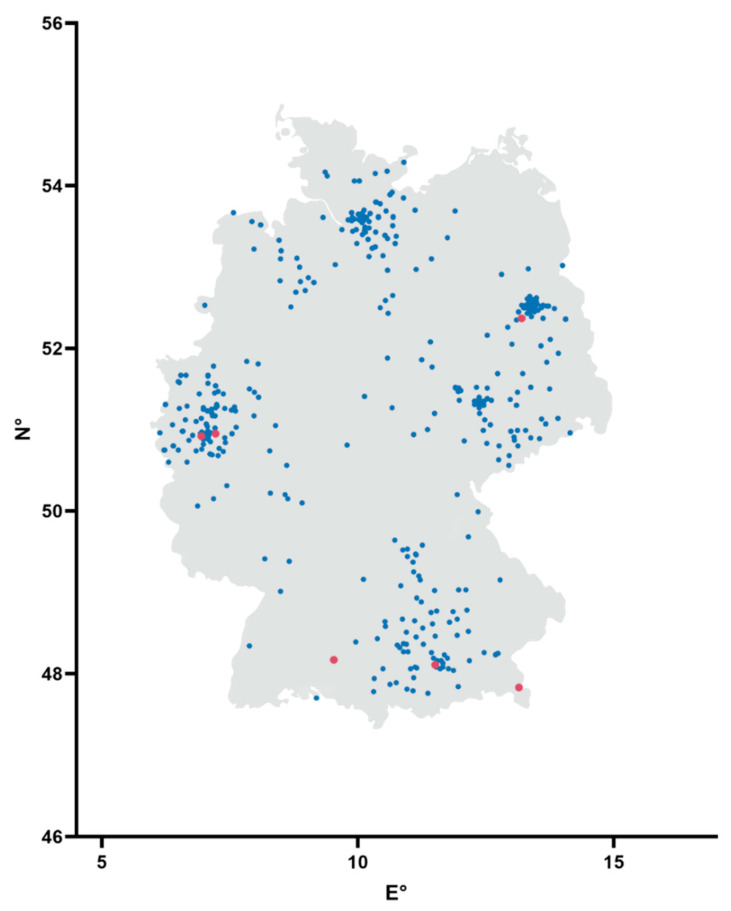
Schematic map of Germany in a coordinate system. Blue points indicate negative samples and red points indicate samples that were found to be positive for antibodies against SARS-CoV-2 by ELISA and an indirect immunofluorescence test (iIFT). N° = decimal degrees of longitude, and E° = decimal degrees of latitude.

In total, 920 cat serum samples were obtained from Synlab, a company in Augsburg, Germany, which is a provider for veterinary diagnostics. The samples were taken from domestic pets during clinical examination by the attending veterinarian and sent to Synlab for hematology testing. Therefore, information about the health status of the cats and their respective owner is not available. The samples were collected from April to September and originated from all over the country (Figure 1). The sample origin covered 15 of the 16 German federal states. For details about the sample number per federal state, see Table 1.

### 2.2. Antibody Detection

Serum samples were screened for antibodies against SARS-CoV-2 using an already validated indirect multi-species ELISA based on the receptor-binding domain (RBD) of SARS-CoV-2 [20]. In short, serum samples were diluted 1/100 and incubated simultaneously in a coated and uncoated well (50 µL per well). After washing with Tris-buffered saline with Tween 20 (TBST), a multi-species conjugate (SBVMILK; IDvet, Grabels, France) was added and incubated for another hour. Following another washing step, tetramethylbenzidine (TMB) substrate (IDEXX, Liebefeld, Switzerland) was added and ten minutes later, the reaction was blocked by adding a stop solution (IDEXX, Liebefeld, Switzerland). Reading was performed at a wavelength of 450 nm on a Tecan Spectra Mini instrument (Tecan Group Ltd., Männedorf, Switzerland). The absorbance was calculated by subtraction of the optical density (OD) value of the uncoated well from the OD value of the coated well. Absorbance of ≥0.3 was defined as positive and values of ≤0.2 were defined as negative by prior validation [20]. The intermediate zone was seen as inconclusive and described as borderline. For confirmation, a subset of 77 ELISA-negative samples were additionally tested by an indirect immunofluorescence assay (iIFA) against SARS-CoV-2. All samples that reacted positive or borderline in the RBD-ELISA were likewise tested by iIFA and additionally investigated for neutralizing antibodies against SARS-CoV-2 by a virus neutralization test (VNT). Both tests have been described by Schlottau et al. [21]. In short, VNT was performed in triplicate by incubating serum samples with a tissue culture infectious dose 50 (TCID_50_) of 10^3.3^ per mL of the 2019_nCoV Muc-IMB-1 SARS-CoV-2 isolate, and adding Vero E6 cells (L 0929, Collection of Cell Lines in Veterinary Medicine (CCLV), Friedrich-Loeffler-Institut, Greifswald—Insel Riems, Germany) after one hour of incubation. The read-out was the cytopathic effect after 3 days, which was expressed as the dilution that caused 50% neutralization (ND_50_). For the indirect immunofluorescence test (iIFT), confluent Vero cells were infected with a multiplicity of infection (MOI) of 0.1 of SARS-CoV-2 and fixed after 24 h. Serum samples were diluted serially two-fold, starting with 1/8, and added to the cell layer. After one hour, washing was performed and an anti-cat-IgG-FITC (1/600, Sigma-Aldrich, Steinheim, Germany) was added. Finally, another washing step was performed and a fluorescence preservation buffer (Sigma-Aldrich, Steinheim, Germany) was added. Analysis was performed by fluorescence microscopy.

Additionally, all serum samples were screened for neutralizing antibodies against the feline coronavirus (FCoV) by VNT using the isolate “München” (RVB-1259, Virus Collection, Friedrich-Loeffler-Institut, Greifswald—Insel Riems, Germany). The serum samples were serially diluted two-fold, starting with a 1/10 dilution. Testing was performed in duplicate. In total, 50 µL of pre-diluted serum was mixed with 50 µL of medium containing 100 TCID_50_ FCoV and incubated at 37 °C. After two hours, CRFK cells (L 0115, CCLV) were added and incubated for 72 h. Titers are expressed as ND_50_, as determined by the appearance of cytopathic effects. Positive sera were also tested in the iIFT against SARS-CoV-2 to further rule out possible antibody cross-reactivity.

## 3. Results

### 3.1. Detection of Antibodies against SARS-CoV-2

Six of the 920 serum samples (0.69%) tested positive in the ELISA for antibodies against SARS-CoV-2. Another five samples reached the borderline threshold. The positive samples were collected during June and July and originated from three different federal states (Bavaria, Brandenburg, and North Rhine-Westphalia). For details, see Table 1 and Figure 1.

**Figure 2:**
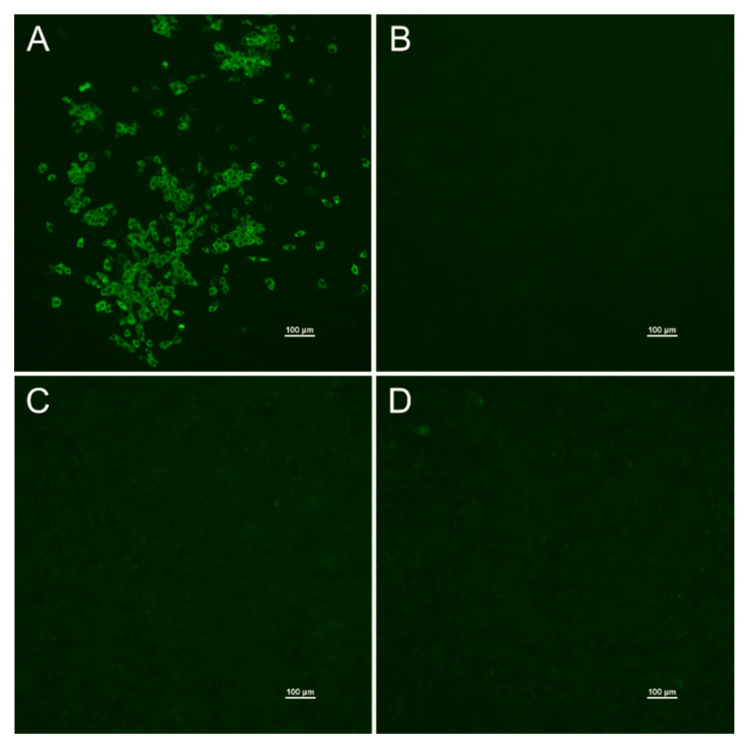
Indirect immunofluorescence assay against SARS-CoV-2. A positive feline serum sample shows a clear fluorescence signal in the virus-infected cell layer (**A**) in contrast to the uninfected cell layer (**B**) that is used as a control. The negative serum sample does not show a fluorescence signal in either the infected (**C**) or uninfected cell layer (**D**). Pictures were taken from 1/256 serum dilutions. Bar is 100 µm.

A subset of 77 ELISA-negative samples were additionally investigated by iIFT and all of them tested negative. All six ELISA-positive samples also tested positive in the iIFA against SARS-CoV-2. Hence, both tests gave concordant results and a clear differentiation between antibody-positive and -negative feline samples was also possible in the iIFT (Figure 2). Two of the serum samples with borderline results in the ELISA tested positive in the iIFT, whilst the other three tested negative. For details, see Table 2.

The VNT detected neutralizing antibodies against SARS-CoV-2 in two of the six samples that reacted positively in the ELISA and iIFT. All borderline samples were investigated by VNT and they tested negative. For details, see Table 2.

### 3.2. Detection of Antibodies against FCoV by VNT

In 25 of the 920 serum samples (2.72%), neutralizing antibodies against the FCoV isolate “München” were detected (Table 3). None of the samples that were positive or had a borderline result in the ELISA against SARS-CoV-2 tested positive for FCoV.

## 4. Discussion

During the ongoing SARS-CoV-2 pandemic, the PCR detection of viral RNA is the main testing system employed to assess the incidence of infections in the human population [22]. This helps in planning and applying quarantine or movement restriction measures, but fails to determine the actual number of people that have been infected with SARS-CoV-2, as asymptomatic infections may be overlooked. Surveys focusing on antibody prevalence have the advantage that infection can be detected even when the causative agent is not present any more. To date, such antibody studies are still rare for the human population of Germany. Therefore, the seroprevalence study of cat sera presented here has to be seen in the context of the overall case numbers, as detected by RT-PCR [23], and the few studies that are already published [24,25].

A first survey of blood donor samples, collected in three federal states of Germany (North Rhine-Westphalia, Lower Saxony, and Hesse) from March to June, describes a seroprevalence of 0.91% (29/3186). Even though this estimated value might be too low due to the age and health conditions of blood donors, it is a first hint of the actual seroprevalence during the first outbreak wave in Germany [25]. Furthermore, a retrospective model estimated that 0.85% of the German population had been infected by the beginning of May, when the first intervention strategies were applied [24]. In this context, this first nationwide surveillance of domestic cats reports a rather high percentage of 0.69% (6/920) serum samples that tested positive for antibodies against SARS-CoV-2. Previous studies on cats from other countries have been conducted with rather small sample sizes and focused on cats from known SARS-CoV-2-positive areas and households. In Wuhan, where the first cases of the novel coronavirus were detected, 14.7% (15/102) of domestic cats, sampled from a local pet hospital during the outbreak, tested positive for antibodies in an RBD-ELISA [12]. Furthermore, a study that was conducted in households with a SARS-CoV-2-positive status in France found a seroprevalence of 23.5% (8/34). If one of the three applied ELISAs would have been counted as antibody-positive against SARS-CoV-2, the prevalence would have been as high as 58.8% (20/34) [13]. Considering that the serum samples in the present study were collected from all over Germany during a period when the incidence of SARS-CoV-2 in Germany was low, the seroprevalence of 0.69% fits with these previous findings of cat populations in risk areas.

On a regional level, the majority of serum samples originated from six federal states. Twenty percent of samples each originated from Saxony and North Rhine-Westphalia, 14% from Berlin, and 11% from Hamburg and Bavaria. Both North Rhine-Westphalia and Bavaria were seen as risk areas during the first SARS-CoV-2 wave in Germany, as they represented 24.14% and 23.50% of all registered human infections at the end of September, respectively [23]. In both federal states, positive feline serum samples were found, with a rate of 1.1% (2/180) in North Rhine-Westphalia and 2.04% (2/98) in Bavaria. In Baden-Wuerttemberg, representing the federal state with the third highest number of registered cases (17.19%), one of five available samples was positive (20.0%). In contrast, no positive samples were detected in Saxony, Berlin, or Hamburg. None of them showed high case numbers, with 2.49%, 2.71%, and 5.01% of all registered human cases, respectively [23]. This shows that the sero-survey of cat sera in Germany corresponds with the virus dispersion in the human population and is a useful tool for assessing the infection level of a certain area. It also indicates that human-to-cat virus transmission is occurring on a regular basis. However, the missing background information about the owners’ SARS-CoV-2 antibody status, occurring clinical symptoms, and living conditions represents a certain limitation of the present study. This information could not be obtained due to private policy regulations and would have added valuable input to the data analysis.

In the present study, serum samples that tested positive against SARS-CoV-2 by an RBD-ELISA were additionally investigated by iIFT and VNT, in order to confirm the antibody status and assess the level of neutralizing antibodies. The iIFT confirmed the antibody-positive status of the pre-screened serum samples, as all of them showed titers ranging between 1/512 and 1/4096. Neutralizing antibodies were only detected in 33.3% (2/6) of the ELISA- and iIFA-positive serum samples and titers of the VNT test did not correspond to the OD values of the ELISA test. This phenomenon was observed in previous studies [12,13] and is most likely due to a delayed production of neutralizing antibodies. Although it has not been demonstrated for cats yet, experimental infection studies on ferrets have shown that neutralizing antibodies appear with a delay of around two weeks after the detection of antibodies by iIFT [21]. An Italian study on domestic cats from SARS-CoV-2-affected areas came to a seroprevalence of 3.95% (6/152) by using only one antibody assay that detects neutralizing antibodies [26]. This rate is considerably higher than the detection rates of the studies that used a combination of at least one ELISA and a neutralization assay [12,13]. In our study, all serum samples with a borderline result from the ELISA screening were tested by iIFT and VNT. While all of them remained negative in the VNT, two serum samples exhibited rather low titers in the iIFT. These serum samples might originate from cats that are in an early stage of viral infection, where neutralizing antibodies are not yet detectable and general antibody levels are still low. Alternatively, these specimens could be taken from cats that were infected some time prior to the sampling, as both were collected at least three months after the first reported human cases in Germany. A first antibody progression study on two cats showed that the detection of neutralizing antibodies is relatively transient and decreases over time [12].

None of the serum samples that displayed neutralizing antibodies against FCoV were positive in the ELISA or the iIFT against SARS-CoV-2. Furthermore, the SARS-CoV-2-positive samples did not show neutralizing antibodies against FCoV. This rules out serologically cross-reactivity of FCoV-specific antibodies with SARS-CoV-2 and confirms results of previous studies, which likewise did not find any cross-reactivity between SARS-CoV-2 and FCoV [12,13]. Hence, the presented sero-prevalence study is not distorted by domestic cats that had come into contact with the FCoV, which is endemic in Germany [27].

Overall, one should keep in mind that the impact of domestic cats on the spread of SARS-CoV-2 during an ongoing high spreading period in humans is not essential. Although around 20% of households in Europe and America are estimated to own a cat as a pet [2], the intensive contact that would be needed for a trans-species spreading event is mostly limited to their owners and is not extended to different households. Therefore, the problem of eventual transmission to and from humans should be evaluated on a smaller scale, such as a household unit [7,28]. As recommended by the European Advisory Board on Cat Diseases (ABCD), cats living in a SARS-CoV-2-infected household should be kept indoors during quarantine and any interactions should be performed under basic hygienic measures [29]. Keeping in mind the current scientific knowledge, there is no indication for drastic measures concerning domestic cats, whether they are free roaming or kept as pets at home.

## 5. Conclusions

This large-scale survey of antibodies against SARS-CoV-2 in the cat population of Germany showed that natural infection is most likely happening on a regular, though infrequent, basis. The incidence of human cases in the country, as well as in the different federal states, reflects the frequency of cats that were infected.

## Figures and Tables

**Table 1:** Number of samples that tested positive in the indirect ELISA against the receptor-binding domain (RBD) of severe acute respiratory syndrome coronavirus 2 (SARS-CoV-2) in the context of all samples taken at a specific time in each federal state of Germany.

Federal State	Month 2020						∑
	April	May	June	July	August	September	
Baden-Wuerttemberg	0/0	0/0	1/2	0/3	0/0	0/0	1/5
Bavaria	0/0	0/39	1/18	1/32	0/0	0/9	2/98
Berlin	0/0	0/40	0/1	0/56	0/30	0/5	0/132
Brandenburg	0/0	0/15	0/0	1/39	0/13	0/1	1/68
Bremen	0/0	0/1	0/1	0/0	0/2	0/0	0/4
Hamburg	0/0	0/16	0/18	0/10	0/36	0/23	0/103
Hesse	0/0	0/0	0/2	0/8	0/2	0/0	0/12
Lower Saxony	0/0	0/13	0/9	0/1	0/7	0/14	0/44
Mecklenburg-Western Pomerania	0/1	0/2	0/0	0/2	0/0	0/2	0/7
North Rhine-Westphalia	0/0	0/16	1/30	1/74	0/58	0/2	2/180
Rhineland-Palatinate	0/0	0/0	0/1	0/1	0/1	0/0	0/3
Saxony	0/19	0/38	0/36	0/46	0/39	0/4	0/182
Saxony-Anhalt	0/2	0/5	0/4	0/8	0/9	0/1	0/29
Schleswig-Holstein	0/0	0/14	0/5	0/4	0/15	0/10	0/48
Thuringia	0/0	0/0	0/1	0/2	0/2	0/0	0/5
∑	0/22	0/199	3/128	3/286	0/214	0/71	6/920

**Table 2:** Detailed information about the results of serum samples that tested positive or reached the borderline threshold in the indirect ELISA against the RBD of SARS-CoV-2. VNT = virus neutralization test, and iIFA = indirect immunofluorescence assay.

Collection Date	RBD ELISA	iIFT SARS-CoV-2	VNT SARS-CoV-2	VNT FCoV
	Absorbance/Result		ND_50_	ND_50_
16 June2020	1.292/positive	1/4096	neg. ^1^	neg. ^1^
7 July 2020	0.850/positive	1/512	1/101.6	neg. ^1^
3 June 2020	0.650/positive	1/1024	neg. ^1^	neg. ^1^
15 July 2020	0.369/positive	1/512	neg. ^1^	neg. ^1^
25 July 2020	0.334/positive	1/1024	1/20.16	neg. ^1^
16 June 2020	0.320/positive	1/2048	neg. ^1^	neg. ^1^
10 September 2020	0.242/borderline	neg. ^1^	neg. ^1^	neg. ^1^
9 September 2020	0.239/borderline	1/512	neg. ^1^	neg. ^1^
9 September 2020	0.225/borderline	neg. ^1^	neg. ^1^	neg. ^1^
8 July 2020	0.207/borderline	1/256	neg. ^1^	neg. ^1^
30 April 2020	0.201/borderline	neg. ^1^	neg. ^1^	neg. ^1^

^1^ neg. stands for a detection limit of <1:8 for the iIFT, <1:16 for VNT SARS-CoV-2, and <1:10 for VNT feline coronavirus (FCoV).

**Table 3:** Number of samples that tested positive in the virus neutralization test against FCoV in the context of all samples taken at a specific time in each federal state of Germany.

Federal State	Month 2020						∑
	April	May	June	July	August	September	
Baden-Wuerttemberg	0/0	0/0	0/2	0/3	0/0	0/0	0/5
Bavaria	0/0	4/39	1/18	1/32	0/0	0/9	6/98
Berlin	0/0	2/40	0/1	0/56	0/30	0/5	2/132
Brandenburg	0/0	0/15	0/0	0/39	1/13	0/1	1/68
Bremen	0/0	0/1	0/1	0/0	0/2	0/0	0/4
Hamburg	0/0	0/16	0/18	0/10	1/36	0/23	1/103
Hesse	0/0	0/0	0/2	1/8	0/2	0/0	1/12
Lower Saxony	0/0	4/13	0/9	0/1	0/7	0/14	4/44
Mecklenburg-Western Pomerania	0/1	0/2	0/0	0/2	0/0	1/2	1/7
North Rhine-Westphalia	0/0	1/16	0/30	1/74	0/58	0/2	2/180
Rhineland-Palatinate	0/0	0/0	0/1	0/1	0/1	0/0	0/3
Saxony	0/19	1/38	1/36	2/46	1/39	0/4	5/182
Saxony-Anhalt	0/2	0/5	0/4	0/8	0/9	0/1	0/29
Schleswig-Holstein	0/0	0/14	0/5	1/4	0/15	1/10	2/48
Thuringia	0/0	0/0	0/1	0/2	0/2	0/0	0/5
∑	0/22	12/199	2/128	6/286	3/214	2/71	25/920
